# Epidemiological landscape of androgenetic alopecia in the US: An All of Us cross-sectional study

**DOI:** 10.1371/journal.pone.0319040

**Published:** 2025-02-27

**Authors:** Aditya K. Gupta, Tong Wang, Vasiliki Economopoulos

**Affiliations:** 1 Division of Dermatology, Department of Medicine, University of Toronto School of Medicine, Toronto, Ontario, Canada; 2 Mediprobe Research Inc., London, Ontario, Canada; 3 Department of Medical Biophysics, Schulich Scholl of Medicine and Dentistry, University of Western Ontario, London, Ontario, Canada; Federal University of Minas Gerais: Universidade Federal de Minas Gerais, BRAZIL

## Abstract

**Background:**

Androgenetic alopecia (AGA) is extremely prevalent with a multifactorial etiology.

**Materials:**

We conducted a cross-sectional study using the All of US (AoU) dataset Sept 2024 to better understand the epidemiology, social determinants and management of AGA.

**Results:**

Most males were 20–39 years old and females 60–69 years old. Men typically have an earlier onset of AGA than females. Male AGA is generally managed with finasteride; oral minoxidil is prescribed in younger males. Females are prescribed spironolactone and oral minoxidil with finasteride in post-menopausal females. There was very little dutasteride prescribed. Topical minoxidil is available over the counter and was not evaluated. Early in 2011 there were reports of the Post-Finasteride Syndrome (PFS); subsequently, the finasteride prescription rate fell to about 10–20% of the pre-PFS prescription rate. There was increased reporting for AGA in those who drink, have an annual household income ≥$75,000, and those with a higher level of education. There was also higher reporting of female AGA in those with anxiety and depression. Patients with higher income and education may have less pressing medical concerns enabling them to bring their AGA to the physician’s attention. Females in whom the AGA affects their anxiety and depression may seek help for the AGA as a way to address their underlying disorder.

**Conclusions:**

This study provides a snapshot of the epidemiology and management of AGA in the USA. AGA is linked to the social determinants of health; addressing the AGA may help better manage the underlying mental and physical state.

## Introduction

Androgenetic alopecia (AGA) is the most common form hair loss, with up 80% of men and 50% of women developing AGA by the age of 70 [1–3]. Even though AGA does not lead directly to significant mortality or morbidity, it can significantly alter a person’s appearance, which can lead to significant psychological impact [4–6]. This disorder can be especially detrimental to women’s psychological well-being, reducing self-esteem and confidence, and possibly leading to added anxiety and depression [6].

In addition to the psychological impact, AGA is also associated with various comorbid conditions, including metabolic, endocrine and thyroid disorders, with potentially a portion of cases occurring secondary to these conditions [7,8].

Considering the above factors and that AGA is an extremely common condition, it may be an indicator of patient overall health. However, since AGA is so common there may be differences in management which depend on different demographic factors, potentially also making AGA an indicator of patient access to dermatological healthcare.

Disparities in access to dermatological healthcare are already known to exist. Several groups have described the challenges of treating under-insured and uninsured patients, as well as having detailed the underlying causes for some disparities that currently exist. [9–11]

Databases like the All of Us (AoU) research program offer the unique opportunity to examine how the care for a particular condition may be impacted not only by concurrent conditions, but also by specific demographic factors. The AoU program is an initiative created by the National Institutes of Health (NIH) which consists of survey, electronic health records, physical measurements and genomic data for individuals enrolled within the program. This data is then made available to researchers through data usage and security agreement negotiated with individual institutions, including – but not limited to – universities and hospital research facilities [12–14].

In this work, we conducted a retrospective cross-sectional study to examine treatment trends and prevalence of AGA diagnoses within the AoU research program, focusing on differences between demographic groups as well as between different concurrent conditions.

## Methods

We assessed data from the All of Us (AoU) research program, which aims to provide diverse and inclusive health data for medical research. All analyses were performed using the registered tier dataset version 7 available within the AoU research program, which includes data collected from US participants between the summer of 2017 and July 1, 2022. This dataset includes electronic health records collected from health care providers and survey data for individuals that have volunteered to participate in the program. Additional information regarding the AoU research program can be found in the indicated references [12–14]. Specific ethics approvals for this study were not required as the NIH has obtained prior approvals for the AoU program, with data usage agreements in place with partnered institutions. Researchers affiliated with partnered institutions are required to complete privacy and data security training before access to the dataset is provided. Additionally, no patient identifiers were collected. S1 and S2 Files contain the STROBE/RECORD Checklist and the PLoS one Human Research Subject Checklist.

Only participants with available electronic health records as well as completed survey questions of interest for demographic factors were selected to be included in this study (n =  266,612). No further exclusions were made. We assessed the incidence/reporting of androgenic alopecia (AGA) in both male and female patients separately. We examined the age distribution of AGA patients as well as the proportion of patients at each age bracket that received pharmacological treatment.

Using the concept IDs found within the Observational Medical Outcomes Partnership (OMOP) framework, and searchable through the Athena OHDSI Vocabularies Repository (Odysseus Data Services, Inc.), we identified patients with AGA as well as comorbid conditions and prescription treatments for minoxidil, finasteride, and spironolactone (female patients only). Using the available survey data, we examined the incidence/reporting of AGA by the following demographic factors: alcohol use, income (less than VS greater than $75,000/year) and education (less than VS greater than Bachelor’s degree). We also investigated the incidence of AGA according to the following comorbidities in females: polycystic ovarian syndrome (PCOS), anxiety, depression, bipolar disorder, and post-traumatic stress disorder (PTSD) and obesity by body mass index (BMI) category. S1 Table lists all concept ID codes that were used in this study.

In order to uphold the privacy of participants in the AoU research program, we have presented all data within graphs as percentages where applicable. We have also replaced any patient numbers within tables that are less than 20 with a value of < 20 in accordance with AoU guidelines. N-values are listed within each table shown in this manuscript. Summary statistics for AGA populations can be found within S2 Table.

The SAS Studio application in the Researcher Workbench environment within the AoU portal was used to perform all analyses. Logistic regression and Wald’s chi-square tests were used for the statistical analysis, with the unadjusted odds ratios calculated for each sex independently. The Cochran-Armitage test was used to assess prescription trends. The odds ratio (OR) was calculated for each comparison and we set the significance level to a =  0.05. We used the Benjamini-Hochberg method with a false discovery rate (Q) of 0.1 to account for multiple comparisons.

## Results

### Age distribution of AGA and prescription medications

**Fig 1:**
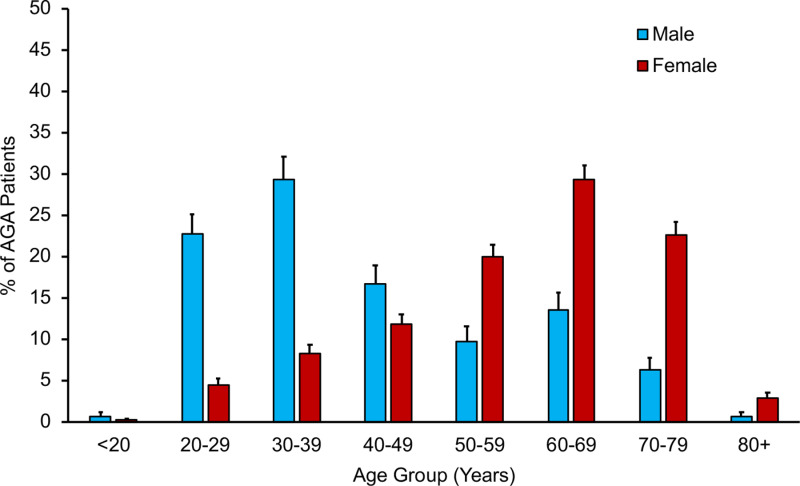
Age distribution of male (n =  286) and female AGA patients (n =  706).

We first examined the age distribution of AGA patients (Fig 1). The incidence/reporting of AGA in males tends to occur at a younger age (22.73% for 20–29 years and 29.37% for 30–39 years, p <  0.0001) and at an older age for females (29.32% for 60–69 years, p <  0.0001).

**Fig 2:**
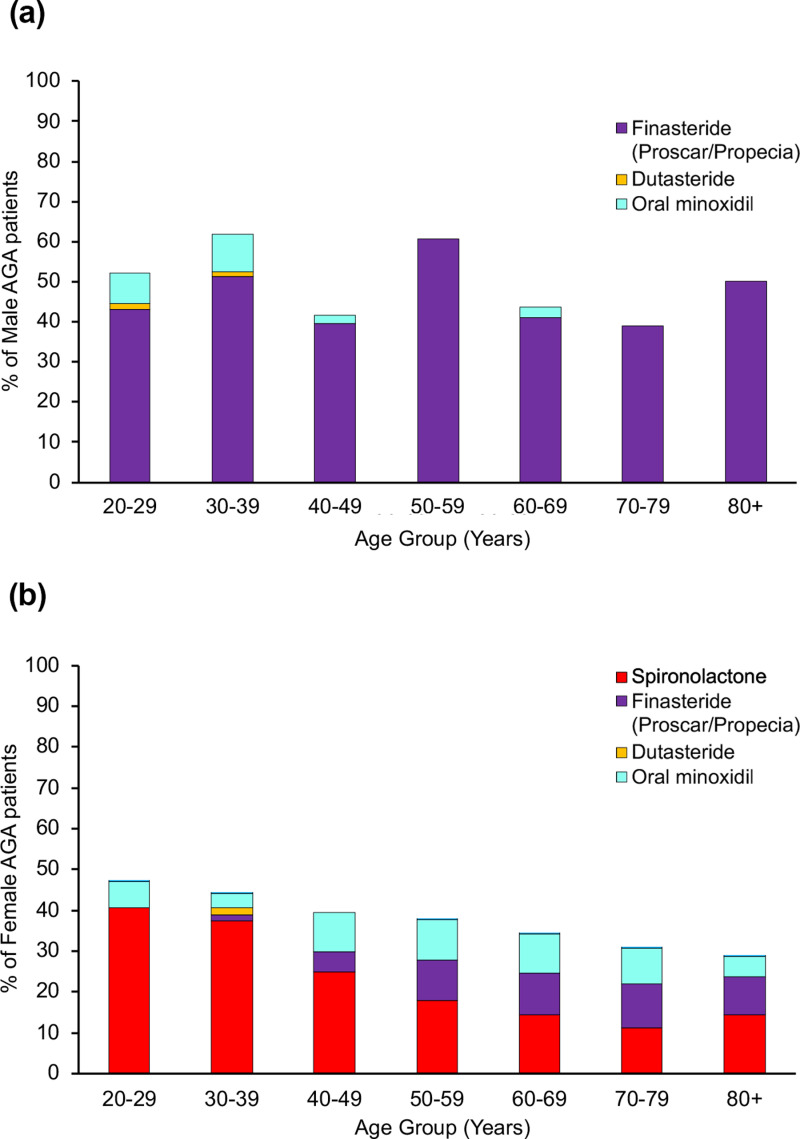
Age distribution of AGA patients receiving prescription treatment. (A) Male AGA patients (n =  286); (B) female AGA patients (n =  706).

We next looked at the distribution of patients receiving prescription medications. Fig 2A and 2B show the proportion of AGA patients in each age group receiving finasteride and oral minoxidil (off-label) for males with spironolactone, oral minoxidil and finasteride (all off-label) for women. There were essentially no prescriptions for dutasteride to treat AGA. We did not look for topical minoxidil since it can be purchased over the counter.

In male patients (Fig 2A) finasteride was the predominant medication, ranging from 38.9% to 60.7% of patients. The majority of oral minoxidil prescriptions in males with AGA were for the younger age groups (7.69% in 20–29 years, 9.52% in 30–39 years; p =  0.0259). Younger AGA females (Fig 2B) received mostly spironolactone (40.63% of 20–29 years old, 37.29% of 30–39 years old, 25% of 40–49 years old; p =  0.0004), followed by oral minoxidil (6.25% of 20–29 years old, 3.39% of 30–39 years old, 9.52% of 40–49 years old; p < 0.0001), and finasteride (1.69% of 30–39 years old, 4.76% of 40–49 years old; p <  0.0001). In older AGA females ( > 50 years old) the proportion of oral minoxidil (4.76% to 9.93%) and finasteride (9.52% to 10.63%) prescriptions were similar in each age group. Finasteride was prescribed in post-menopausal women since it is contraindicated in pre-menopausal females and those not using appropriate contraception.

### Prescribing trends over time

**Fig 3:**
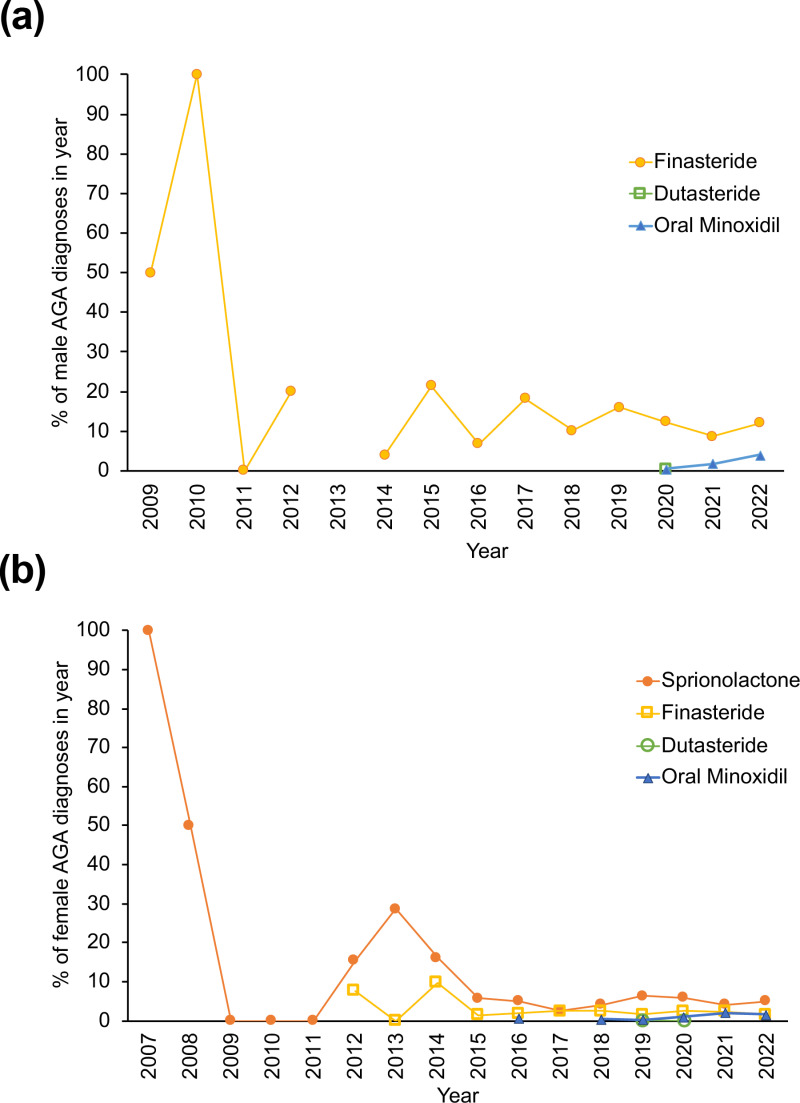
Prescription trends in AGA patients over time. (A) Male AGA patients and (B) female AGA patients.

We examined the prescription patterns of finasteride and oral minoxidil in males and females (Fig 3). In males (Fig 3A), there was a decrease in finasteride usage beginning in 2011, with a drop to approximately 10% to 20% of previous usage, which coincides with the heightened awareness of Post-Finasteride Syndrome (PFS). The trend in finasteride prescriptions was found to be significantly increasing at later years (Cochran-Armitage test: Z-statistic 2.75, p =  0.0060). In females (Fig 3B), the use of spironolactone decreased in 2009 and remained higher than finasteride, dutasteride and oral minoxidil. There was a slight increase in the usage of oral minoxidil starting in 2020 in both males (p =  0.0202) and females (p =  0.0013) when compared to the proportion of diagnosed patients.

### Demographic factors and AGA incidence/reporting

**Fig 4:**
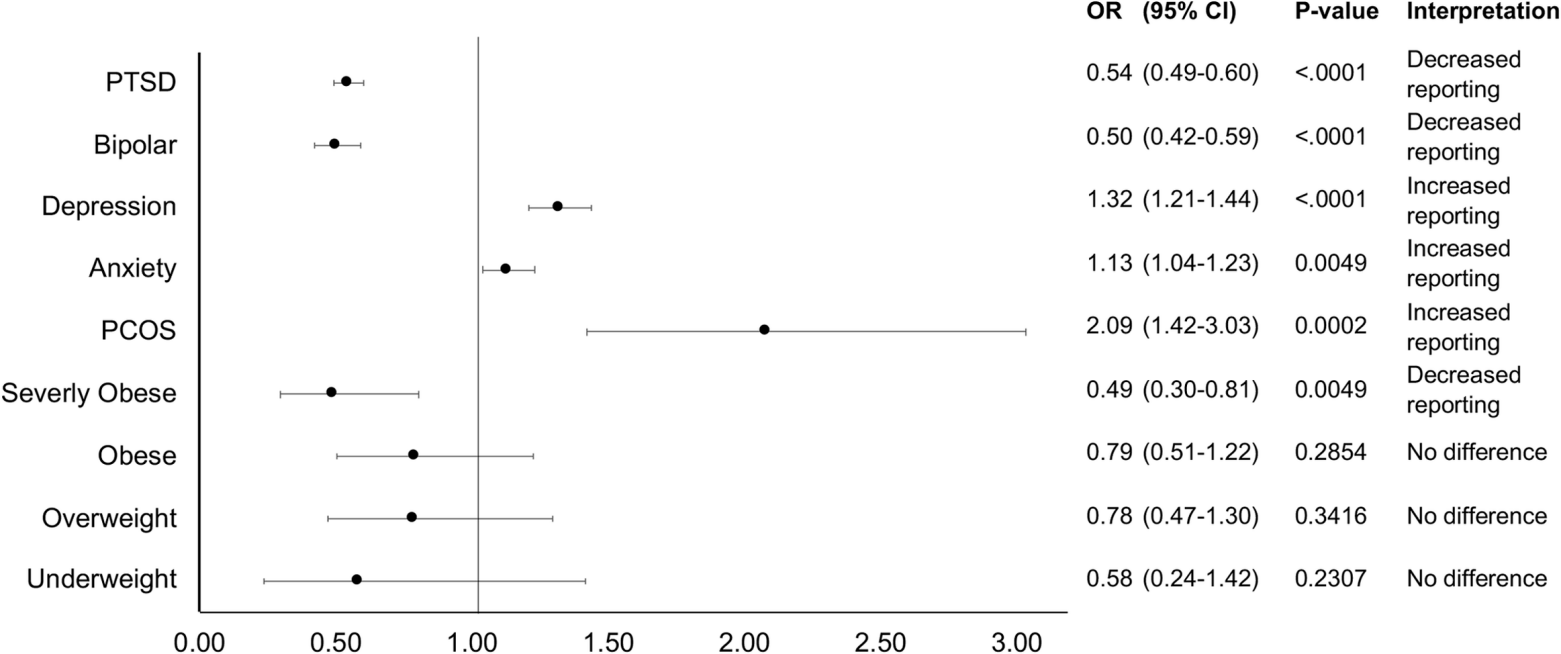
Likelihood of AGA reporting for females by comorbid condition. All data is represented as odds ratios of the condition compared to control with corresponding p-value and interpretation.

The demographic factors – alcohol use, income and education – were studied to determine their association with AGA incidence/reporting, separately for males and females. There was an increase in AGA incidence/reporting for both males and females with alcohol use (Table 1) with odds ratios of 2.11 (1.62 – 2.76) for males (p <  0.0001) and 1.19 (1.01 – 1.39) for females (p =  0.0358). Higher income was associated with increased AGA incidence/reporting (Table 2) in both males (OR 2.51 (1.96 – 3.21), p <  0.0001) and females (OR 1.89 (1.60 – 2.23), p <  0.0001). Higher levels of education were also associated with higher incidence/reporting of AGA (Table 3) in both males and females with odds ratios of 4.32 (3.29 – 5.70) in males (p <  0.0001) and 2.16 (1.85 – 2.52) in females (p <  0.0001). The incidence/reporting of AGA in females was higher in anxiety (OR 1.13 (1.04 – 1.23), p =  0.0049) and depression (OR 1.32 (1.21 – 1.44), p <  0.0001), while being reduced within bipolar disorder (OR 0.50 (0.42 – 0.59), p <  0.0001) and PTSD (OR 0.54 (0.49 – 0.60) p <  0.0001) (Fig 4). There was increased reporting of AGA in females with PCOS (OR 2.09 (1.42 – 3.03), p =  0.0002).

**Table 1:** Reporting of AGA with alcohol use.

	Non drinkers			Drinkers			Drinkers vs. non drinkers		Interpretation
	No AGA	AGA (%)	Total	No AGA	AGA (%)	Total	Odds ratio (CI)	P value	
**Males**	40664	79 (0.19)	40743	42619	175 (0.41)	42794	2.11 (1.62–2.76)	<0.0001	Increased reporting of AGA in drinkers
**Females**	78849	339 (0.43)	79188	57090	291 (0.51)	57381	1.19 (1.01–1.39)	0.0358	Increased reporting of AGA in drinkers

**Table 2:** Reporting of AGA with income.

	High income ($75 K and up)			Low income (less than $75 K)			High vs. low income		Interpretation
	No AGA	AGA (%)	Total	No AGA	AGA (%)	Total	Odds ratio (CI)	P value	
**Male**	30101	159 (0.53)	30260	49353	104 (0.21)	49457	2.51 (1.96–3.21)	<0.0001	Increased reporting of AGA in high income
**Female**	43728	280 (0.64)	44008	83974	284 (0.34)	84258	1.89 (1.60–2.23)	<0.0001	Increased reporting of AGA in high income

**Table 3:** Reporting of AGA with education.

	Higher education (Bachelor’s and up)			Lower education (less than Bachelor’s)			Higher vs. lower education		Interpretation
	No AGA	AGA (%)	Total	No AGA	AGA (%)	Total	Odds ratio (CI)	P value	
**Male**	41817	219 (0.52)	42036	54491	66 (0.12)	54557	4.32 (3.29–5.70)	<0.0001	Increased reporting in AGA in higher education
**Female**	69098	436 (0.63)	69534	89365	261 (0.29)	89626	2.16 (1.85–2.52)	<0.0001	Increased reporting of AGA in higher education

## Discussion

To our knowledge, this is the first study to examine the prevalence of AGA in both male and female participants of different demographic groups and comorbid conditions, as well its treatment in the AoU dataset. The data that we have shown provides an informative view of the AGA management landscape in both men and women in the US.

There are differences in the age of male and female patients that seek medical care for AGA (Fig 1), with men seeking care at younger ages (20–29 and 30–39 years), while women tend to seek care later in life (60–69 years). This distribution is likely due to the differences in presentation of AGA between males and females. AGA has been shown to have different ages of onset in men and women, where AGA affects approximately 30% of men in their 30s, while women tend to experience AGA post-menopause, which is consistent with our observations [6,15].

When examining prescriptions trends, in men with AGA oral finasteride was prescribed at a fairly even rate across all ages (Fig 2A) Oral minoxidil is prescribed more frequently to younger men. In women with AGA younger females were more likely prescribed spironolactone and oral minoxidil (Fig 2B). In older females there was an increase in the prescription rate of finasteride. Finasteride was more likely to be prescribed to women over the age of 50 years, which is consistent with recommendations that 5-ARIs not be prescribed to women who are, or may become pregnant due to risks to the male fetus from impaired DHT signaling [1,15]. Spironolactone is an androgen receptor (AR) antagonist which is commonly used in treating women with polycystic ovarian syndrome (PCOS), which is a known risk factor for AGA [1,16,17]. Treating younger women with spironolactone may be more appropriate than prescribing a 5-ARI, as the risks to a male fetus is lower [1,16].

In male AGA patients, finasteride usage decreased from 2010 onwards, which happens to coincide with increased awareness of PFS. This apparent connection is only speculative, however, Asanad et al. demonstrated through online search data that there was a spike in searches for “finasteride side effects” in 2010 [18]. Its usage had only returned to approximately 20% of pre-PFS levels by 2015 (Fig 3A). In AGA men, the use of oral minoxidil increased from 2020 to 2022, while dutasteride use was minimal. [19,20] In female AGA patients, spironolactone usage was greatest (Fig 3B). Spironolactone acts as an androgen receptor antagonist and is prescribed off-label to female AGA patients. [21] Additionally, this drug is commonly prescribed to women with polycystic ovarian syndrome where up to 42.5% of these patients will experience AGA, which may contribute to the higher levels of spironolactone compared to other medications [8].

We analysed the impact of alcohol use, income and education on the reporting of AGA. Those who drink at least 2–3 times per week had increased reporting of AGA for both men and women (Table 1). A relationship between alcohol use and AGA has not been thoroughly studied. Only a small number of studies have been conducted that examined the relationship, with one study observing an increased risk of AGA in men aged 40 to 69 years that consumed alcohol [22]. However, a more recent case-control study, as well as a cross-sectional study, found no difference in the rate of AGA between those that do and do not consume alcohol [23,24].

We next examined income, where we compared those who have an annual household income of more than $75,000 to those that have less (Table 2). Interestingly, we found that in both men and women, there is increased reporting of AGA in those with a higher income. When we next examined education level, comparing the rate of reporting in those with a bachelor’s degree or higher to those without, we found the same result. Both men and women with higher levels of education had increased reporting of AGA (Table 3). This may be occurring because these populations, with higher education and income, have resources to be able to seek care for AGA, whereas those with lower income and education would only be able to prioritize the most important aspects of their health. However, the cut off values that we have set for both education and income, and the simplified breakdown into higher or lower categories may be over-simplified. In reality, income and education will have varying levels which may create a gradient of risk of AGA when compared to these factors. Since reported AGA diagnoses are low compared to the proportion of participants in each level for these factors, using combined levels allows for greater statistical power in the analysis, but will not allow for the greater level of detail that more precise categories could potentially provide.

We examined the impact of various comorbid conditions. In women, there was increased reporting of AGA in PCOS, anxiety and depression (Fig 4). The presence of hair loss may lead them to seek out treatment, especially if the hair loss exacerbates anxiety and depression. Interestingly, preliminary studies on stress have identified several mediators, such as substance P, that inhibits hair growth, indicating a potential causal relationship between psychological parameters and AGA [25]. Pain is a symptom that can reflect an upregulated pro-inflammatory immune response, which is also evident in AGA patients (i.e., “microinflammation”) [26,27].

The retrospective nature of examining patient EHR data that we have presented may provide more information about the impact of various social and health factors on the likelihood that patients seek care. In many of the demographic and health factors that we have presented above, those who are of a higher socio-economic standing or of better health have an increased likelihood of AGA reported within their health records.

This discrepancy may be because these patients have access to more resources, in particular for higher education and income groups. When considering health factors, this may possibly be due to the healthier patients having less pressing medical concerns, enabling some of these patients to seek care with the resources that they have. However, we should note that this is not the case for certain factors. In women with PCOS, thinning hair can be a symptom of the disorder and as such would be brought up during the course of care. In women with anxiety and depression, hair loss may underlie the anxiety and depression of some patients. These patients may then seek out care to help address the underlying AGA to help relieve the stress caused by the pressure to fit within social norms.

There are some limitations to this work. Firstly, EHR data may not always be accurate. A study published by Cai, et al. examined the accuracy of EHR data within the AoU dataset by evaluating the rates of sex incongruent conditions, where it was found that the incongruence rate was less than 1% [28]. Even though this rate seems low, when examining conditions, groups and treatments where the patient numbers are small, this could potentially skew results. Additionally, Bell et al. found in their study of patient-reported errors in EHR data that 21.1% of patients were able to identify errors within their health data. These types of errors have the potential to skew results of any retrospective study of EHR data [29]. Another limitation of this work is that the specialty of the provider is not available to researchers that have access to the AoU dataset. This information is suppressed in an effort to protect patient privacy. Practitioners in different specialties will have different approaches to treating AGA, with dermatologists possibly being more likely to prescribe medications for AGA patients, while a general practitioner may not. By not having this information, we cannot determine how AGA reporting and prescribing practices vary between specialties.

We have also chosen not to include topical minoxidil within our study due to its over-the-counter availability and potential under-reporting in the AoU dataset. Over-the-counter medications play a significant role in the treatment of many different ailments [30,31], and by not capturing this aspect of care, we risk under-estimating the extent of AGA treatment. Currently, combined finasteride, dutasteride and oral minoxidil usage in males ranges from 38.89% to 61.90% for depending on age, but these values would be higher if topical minoxidil was included, however this would still under-estimate usage as not all topical minoxidil usage is captured within the AoU dataset.

Additionally, some of the treatment options available to patient for hair loss associated with AGA may not be recorded within the AoU dataset. Many of the procedures that a patient may undergo are considered cosmetic (e.g., platelet rich plasma, low level laser therapy and scalp micropigmentation) and not coded within EHR data. A patient may seek initial care through their primary care provider or a dermatologist, but may then attend a different practice/clinic for cosmetic treatment.

Lastly, while we analysed males and female separately, we have not specifically adjusted for age an in some instances were only able to conduct our analysis for only one factor at a time. By not adjusting for all possible factors at the same time there is a small risk that the impact of some of these factors is over-estimated.

## Conclusion

To the best of our knowledge, this is the first study to examine the rate of AGA reporting in the AoU dataset. Our work points to differences in AGA reporting which highlights the influence of social determinants of health. Even though AGA does not significant mortality and morbidity to patients, its level of reporting has the potential to be an important indicator of access inequities within a health care system.

## Supplementary information

S1 Table Concept IDs used with the All of Us dataset. (DOCX)

S2 Table Summary Statistics for AGA populations; populations with less than 20 participants are listed as ‘<20’ to protect patient privacy in accordance with All of Us privacy regulations. (DOCX)

S1 File STROBE/RECORD Checklist. (DOCX)

S2 File PLoS one human research subject checklist. (DOCX)
