# Supplementary material for: Epidemiological landscape of androgenetic alopecia in the US: An All of Us cross-sectional study
Source: PLoS One. 2025 Feb 27;20(2):e0319040. doi: 10.1371/journal.pone.0319040 (PMC11867384; doi:10.1371/journal.pone.0319040)
Supplement: S1 Table — (DOCX) [file pone.0319040.s001.docx]

**Table S1** – Concept IDs used with the All of Us dataset

| **Concept ID** | **Concept Name** |
| --- | --- |
|  |  |
| 19023231 | finasteride 1 MG Oral Tablet |
| 19077375 | finasteride 5 MG Oral Tablet |
| 19025985 | finasteride 5 MG Oral Tablet [Proscar] |
| 996416 | finasteride |
| 996419 | finasteride 1 MG Oral Tablet [Propecia] |
| 19120262 | finasteride 1 MG [Propecia] |
| 989485 | dutasteride 0.5 MG Oral Capsule |
| 989486 | dutasteride 0.5 MG Oral Capsule [Avodart] |
| 989482 | dutasteride |
| 1309068 | minoxidil |
| 1309069 | minoxidil 10 MG Oral Tablet |
| 1309070 | minoxidil 2.5 MG Oral Tablet |
| 19025147 | minoxidil 10 MG Oral Tablet [Loniten] |
| 19025146 | minoxidil 2.5 MG Oral Tablet [Loniten] |
| 1309098 | minoxidil 20 MG/ML Topical Solution |
| 36026925 | minoxidil 20 MG/ML Topical Solution [Regoxidine] |
| 19035384 | minoxidil 20 MG/ML Topical Solution [Rogaine] |
| 1309126 | minoxidil 50 MG/ML Topical Foam |
| 19126186 | minoxidil 50 MG/ML Topical Foam [Rogaine] |
| 1309099 | minoxidil 50 MG/ML Topical Solution |
| 1536908 | minoxidil 50 MG/ML Topical Solution [Regoxidine] |
| 19132621 | minoxidil 50 MG/ML Topical Solution [Rogaine] |
|  |  |
|  |  |
| 970250 | spironolactone |
| 970282 | spironolactone 100 MG Oral Tablet |
| 19023867 | spironolactone 100 MG Oral Tablet [Spirolone] |
| 19079658 | spironolactone 25 MG Oral Tablet |
| 19023830 | spironolactone 25 MG Oral Tablet [Aldactone] |
| 19036703 | spironolactone 25 MG Oral Tablet [Spironol] |
| 970283 | spironolactone 50 MG Oral Tablet |
| 19023827 | spironolactone 50 MG Oral Tablet [Aldactone] |
| 19023828 | spironolactone 50 MG Oral Tablet [Spiroctan] |
|  |  |
| 1586203 | Drink Frequency Past Year: Monthly Or Less |
| 1586204 | Drink Frequency Past Year: 2 to 4 Per Month |
| 1586202 | Drink Frequency Past Year: Never |
| 1586205 | Drink Frequency Past Year: 2 to 3 Per Week |
| 1586206 | Drink Frequency Past Year: 4 or More Per Week |
|  |  |
| 1585634 | 6 or More Drinks Occurrence: Never In Last Year |
| 1586214 | 6 or More Drinks Occurrence: Less Than Monthly |
| 1586215 | 6 or More Drinks Occurrence: Monthly |
| 1586216 | 6 or More Drinks Occurrence: Weekly |
| 1586217 | 6 or More Drinks Occurrence: Daily |
|  |  |
| 1586208 | Average Daily Drink Count: 1 or 2 |
| 1586209 | Average Daily Drink Count: 3 or 4 |
| 1586210 | Average Daily Drink Count: 5 or 6 |
| 1586211 | Average Daily Drink Count: 7 to 9 |
| 1586212 | Average Daily Drink Count: 10 or More |
|  |  |
| 4004847 | Female pattern alopecia |
| 4339092 | Male pattern alopecia |
|  |  |
| 4135421 | Body mass index 20-24 – normal |
| 4060705 | Body mass index 25-29 – overweight |
| 4060985 | Body mass index 30+ - obesity |
| 4256640 | Body mass index 40+ - severely obese |
| 4147565 | Body mass index less than 20 |
| 4241223 | Hidradenitis suppurativa |
| 836850 | Including yourself, who in your family has had anxiety reaction/panic disorder? Select all that apply. – Select patients with “Self” |
| 836803 | Including yourself, who in your family has had bipolar disorder? Select all that apply. – Select patients with “Self” |
| 836804 | Including yourself, who in your family has had depression? Select all that apply. – Select patients with “Self” |
| 1740562 | Including yourself, who in your family has had post-traumatic stress disorder (PTSD)? Select all that apply. – Select patients with “Self” |
| 40443308 | Polycystic ovarian syndrome |
|  |  |
| 1585946 | Highest Grade: College One to Three |
| 1585947 | Highest Grade: College Graduate |
| 1585948 | Highest Grade: Advanced Degree |
| 2000000006 | College Grad and Higher |
| 2000000007 | Less Than High School |
| 1585945 | Highest Grade: Twelve Or GED |
| 1585943 | Highest Grade: Five Through Eight |
| 1585944 | Highest Grade: Nine Through Eleven |
| 1585942 | Highest Grade: One Through Four |
| 1585941 | Highest Grade: Never Attended |
|  |  |
| 1585376 | Annual Income: less 10k |
| 1585377 | Annual Income: 10k 25k |
| 1585378 | Annual Income: 25k 35k |
| 1585379 | Annual Income: 35k 50k |
| 1585380 | Annual Income: 50k 75k |
| 1585381 | Annual Income: 75k 100k |
| 1585382 | Annual Income: 100k 150k |
| 1585383 | Annual Income: 150k 200k |
| 1585384 | Annual Income: more 200k |
|  |  |
|  |  |
|  |  |
