# Supplementary material for: Epidemiological landscape of androgenetic alopecia in the US: An All of Us cross-sectional study
Source: PLoS One. 2025 Feb 27;20(2):e0319040. doi: 10.1371/journal.pone.0319040 (PMC11867384; doi:10.1371/journal.pone.0319040)
Supplement: S2 Table — (DOCX) [file pone.0319040.s002.docx]

**S2 Table. Summary Statistics for AGA populations**; populations with less than 20 participants are listed as ‘<20’ to protect patient privacy in accordance with All of Us privacy regulations

| **Item** |  | **Proportion of AGA patients (n; %)** |
| --- | --- | --- |
| *Number of patients* |  |  |
|  | Male | 286; 28.8% |
|  | Female | 706; 71.2% |
|  |  |  |
| *Prescriptions* |  |  |
|  |  |  |
|  | Finasteride | 192; 19.4% |
|  | Dutasteride | <20; <7% |
|  | Oral Minoxidil | 76; 7.7% |
|  | Spironolactone | 132; 18.7% |
|  |  |  |
|  |  |  |
| *Comorbidity* |  |  |
|  | PTSD | <20; <3% |
|  | Bipolar | <20; <3% |
|  | Depression | 47; 6.7% |
|  | Anxiety | 74; 10.5% |
|  | PCOS | 28; 4.0% |
|  | Severely Obese | 44; 6.2% |
|  | Obese | 117; 16.6% |
|  | Overweight | 42; 5.9% |
|  | Underweight | <20; <3% |
|  |  |  |
| *Demographic factors* |  |  |
|  |  |  |
|  | Drinkers | 418; 42.1% |
|  | High level of education  (Bachelor’s degree or higher) | 655; 66.0% |
|  | High income (> $75,000/year) | 439; 44.3% |
